# Extending analytic methods for economic evaluation in implementation science

**DOI:** 10.1186/s13012-022-01192-w

**Published:** 2022-04-15

**Authors:** Meghan C. O’Leary, Kristen Hassmiller Lich, Leah Frerichs, Jennifer Leeman, Daniel S. Reuland, Stephanie B. Wheeler

**Affiliations:** 1 Department of Health Policy and Management, Gillings School of Global Public Health, University of North Carolina, Chapel Hill, NC USA; 2 Lineberger Comprehensive Cancer Center, University of North Carolina, Chapel Hill, NC USA; 3 School of Nursing, University of North Carolina, Chapel Hill, NC USA; 4 Division of General Medicine & Clinical Epidemiology, School of Medicine, University of North Carolina, Chapel Hill, NC USA

**Keywords:** Economic evaluation, Implementation science, Costs and cost analysis, Colorectal cancer screening

## Abstract

**Background:**

Economic evaluations of the implementation of health-related evidence-based interventions (EBIs) are conducted infrequently and, when performed, often use a limited set of quantitative methods to estimate the cost and effectiveness of EBIs. These studies often underestimate the resources required to implement and sustain EBIs in diverse populations and settings, in part due to inadequate scoping of EBI boundaries and underutilization of methods designed to understand the local context. We call for increased use of diverse methods, especially the integration of quantitative and qualitative approaches, for conducting and better using economic evaluations and related insights across all phases of implementation.

**Main body:**

We describe methodological opportunities by implementation phase to develop more comprehensive and context-specific estimates of implementation costs and downstream impacts of EBI implementation, using the Exploration, Preparation, Implementation, Sustainment (EPIS) framework. We focus specifically on the implementation of complex interventions, which are often multi-level, resource-intensive, multicomponent, heterogeneous across sites and populations, involve many stakeholders and implementation agents, and change over time with respect to costs and outcomes. Using colorectal cancer (CRC) screening EBIs as examples, we outline several approaches to specifying the “boundaries” of EBI implementation and analyzing implementation costs by phase of implementation. We describe how systems mapping and stakeholder engagement methods can be used to clarify EBI implementation costs and guide data collection—particularly important when EBIs are complex. In addition, we discuss the use of simulation modeling with sensitivity/uncertainty analyses within implementation studies for projecting the health and economic impacts of investment in EBIs. Finally, we describe how these results, enhanced by careful data visualization, can inform selection, adoption, adaptation, and sustainment of EBIs.

**Conclusion:**

Health economists and implementation scientists alike should draw from a larger menu of methods for estimating the costs and outcomes associated with complex EBI implementation and employ these methods across the EPIS phases. Our prior experiences using qualitative and systems approaches in addition to traditional quantitative methods provided rich data for informing decision-making about the value of investing in CRC screening EBIs and long-term planning for these health programs. Future work should consider additional opportunities for mixed-method approaches to economic evaluations.

Contributions to the literature

- Economic evaluations of evidence-based intervention (EBI) implementation are scarce and often exclude relevant costs and effects. To address these gaps, we describe mixed-method approaches to estimating costs and benefits of EBI implementation across implementation phases.
- We highlight the particular need for integration of quantitative and qualitative approaches to conducting economic evaluations when implementing complex EBIs, in which careful consideration of the context, perspectives, and balance of required resources and health impact is needed.
- Practical examples of how diverse methods have been applied to cost analyses of complex colorectal cancer screening EBIs are provided to guide future economic evaluations.

## Background

There has been a growing call over the past decade for economic evaluations of evidence-based intervention (EBI) implementation within public health [1–5]. Applying economic evaluations to implementation research involves comparing the costs (e.g., labor, materials) required to support EBI adoption, implementation, and sustainability [1]. Relevant outcomes for implementation research may include intervention reach (i.e., proportion of target population receiving the EBI), fidelity (i.e., adherence to how the EBI was intended to be implemented), and effectiveness (e.g., EBI’s ability to positively affect health outcomes), all of which can be incorporated into economic evaluations (e.g., cost per person reached by an intervention) [1, 5]. Economic evaluations are essential to providing diverse decision-makers with meaningful data about the economic and programmatic feasibility of investing in EBIs across contexts, which implementation strategies work well where and under what circumstances, resources required upfront and over time to execute these strategies, and expected downstream gains (e.g., cost-savings, improved health outcomes) [3, 5]. These data are critical to securing buy-in to implement EBIs [6] and appropriately planning for implementation with respect to costs and resources to support the EBI’s adoption and sustainment [3, 4].

The quantity and quality of economic evaluations in implementation research have improved over time [2]; however, these analyses remain scarce [2, 5, 7–9]. Existing studies commonly lack sufficient detail about the costs associated with implementing new interventions, lack justification for the analytic methods used, and rely on data collected retrospectively after implementation has occurred [2, 8]. While existing implementation frameworks have acknowledged the importance of economic factors to the field, they typically provide little guidance on how to collect and analyze cost-related data [10].

Another concern is that economic evaluations have often used strictly quantitative approaches to estimate the value of EBIs. Adding qualitative approaches to these studies has potential to address quantitative data limitations [1, 5, 11, 12]. Dopp and colleagues identified mixed-method opportunities to understand how stakeholders *across settings and perspectives* interpret findings about implementation costs and cost-effectiveness results (e.g., whether costs collected represent their perspective or seem reasonable), and how implementation resources vary depending on existing infrastructure (e.g., something may or may not have been a cost *to them* because it was or was not in place) [1]. Due to the large absence of these approaches in prior economic evaluations, questions remain about how to effectively use mixed methods across implementation phases to understand variation in costs by context and inform the projection of downstream costs and outcomes [1]. Robust discussion of the value of these methods in informing EBI implementation, adaptations, and/or sustainability is also missing.

In this paper, we argue that mixed-method approaches should be used to conduct economic evaluations in implementation research, with attention to each phase of the implementation process. We recommend a broader range of analytic methods to develop comprehensive and context-specific estimates of the costs and long-term impacts of EBI *implementation.* Given that they are particularly challenging and context-dependent, we focus on evaluation of complex EBIs [13–15], which are commonly multicomponent and multi-level, use multiple implementation strategies, engage diverse stakeholders and implementation agents at all levels (e.g., quality improvement teams, health management executives, population health managers, clinicians, clinic administrative staff, etc.), and require coordination across systems. There can be substantial heterogeneity in costs and resources required across sites, across populations, and by perspective, and the associated costs and benefits may accrue at different time periods during implementation. The context drives important differences in the types of resources needed and the frequency and intensity with which those resources and health outcomes should be estimated. We provide examples of how we have applied the described methods to analyses of multicomponent colorectal cancer (CRC) screening interventions in two studies.

## Main text

### Framework

Guided by the Exploration, Preparation, Implementation, Sustainment (EPIS) framework, we demonstrate how diverse analytic methods can be integrated and applied to economic analyses. The EPIS framework describes implementation research as occurring across four phases: (1) Exploration, which involves EBI selection to address the problem and fit the context; (2) Preparation, which involves designing implementation strategies to integrate EBIs into practice; (3) Implementation, during which EBIs and implementation strategies are initiated and evaluated; and (4) Sustainment, during which EBIs are institutionalized [16, 17]. While we selected EPIS because of its wide use [16] and comprehensive set of economic-related constructs [10], other implementation frameworks [18, 19] propose similar phases of intervention implementation and could also be used to guide economic evaluations. It is most important to identify and estimate EBI implementation costs and benefits and inform decisions across all implementation phases, as studies have often focused on Implementation phase activities [16]. Table 1 shows how EPIS can be used to consider the costs and benefits of conducting economic evaluations of EBI implementation by phase; true to the framework of cost-effectiveness analysis, we present examples of the resources expended to conduct these analyses (i.e., the costs) and examples of what is gained by performing economic evaluations, such as information to improve EBI implementation and outcomes (i.e., the benefits).

**Table 1:** Expected resource requirements and potential benefits of conducting economic evaluations of intervention implementation by implementation phase

EPIS Phase	What resources or efforts may be expended when conducting economic evaluations at each phase of implementation?	What can be gained by conducting economic evaluations at each phase of implementation?
Exploration	• Reflect on insights from intervention studies in other contexts. Identify the similarities and differences to the local context. • Assess organization’s priorities, available resources, and outcomes of interest.	• Assess evidence from other implementation studies and economic evaluations to support decision-making about which EBIs and implementation strategies to adopt for the target population and setting. • Obtain an initial list of relevant costs and resources to include in economic evaluation from prior studies.
Preparation	• Determine what usual care looks like and what the intervention will need to include (e.g., which implementation strategies will work). • Identify boundaries of the intervention. • Map cost data collection activities onto systems maps to guide methods for estimating costs. • Document resources needed to accomplish upfront tasks (training, development of electronic health record systems, etc.). • Consider who and what is needed to successfully implement the intervention.	• Assess evidence from other implementation studies and economic evaluations to select and tailor implementation strategies to optimize return on investment for intended EBI, population, and setting. • Gain an understanding of potential “voltage drops” (i.e., process steps that reduce the overall effectiveness of the intervention) and which resources and costs may be required to address these gaps. • Develop a comprehensive list of cost and fidelity measures to collect and evaluate during the implementation phase.
Implementation	• Track all cost and fidelity measures on a regular basis. • Ask implementation agents about their resource adequacy, time spent on specific activities, and suggestions for improvement. • Streamline tasks based on understanding and specificity of the intervention boundaries. • Consider possible adaptations.	• Gather data on cost and fidelity measures collected on an ongoing basis during intervention implementation that will inform the economic evaluation. • Estimate the time required to implement each step of the intervention, including labor-intensive activities. • Obtain feedback from key stakeholders (patients, implementation agents, decision-makers, etc.) on the costs and benefits of the intervention.
Sustainment	• Estimate the short-term and long-term cost-effectiveness of the intervention. Evaluate the relative impact of uncertainties on the overall cost-effectiveness. • Continue to support anyone with a task in implementing the complex intervention. Identifying who has to do what and what is needed to do it well will help others in the Exploration phase. • Conduct ongoing assessment to identify any changes over time in the resource requirement and the impact on costs.	• Generate evidence to support decision-making on adaptation and sustainment of the intervention. • Estimate the cost-effectiveness of the intervention compared to other alternatives for the outcomes of interest. • Gather input from key stakeholders on how to create economies of scale.

### Case studies

To illustrate how mixed-method approaches can be used to inform implementation economics [20], we describe our experiences using these approaches in two research studies assessing the implementation costs and outcomes of complex CRC screening interventions. The first study is Scaling Colorectal Cancer Screening Through Outreach, Referral, and Engagement (SCORE), a pragmatic randomized trial comparing the effectiveness of mailed fecal immunochemical testing (FIT) and patient navigation to diagnostic colonoscopy, versus usual care, in improving CRC screening among North Carolina community health center (CHC) patients [21]. The implementation strategies to support the FIT intervention include, but are not limited to, developing and managing a centralized clinical CRC screening registry, creating a mailed FIT outreach center, and conducting cycles of intervention testing and adaptation. Strategies are employed by staff in a centralized outreach center in collaboration with CHC clinicians and administrative staff. SCORE is being conducted as part of the National Cancer Institute (NCI)-funded consortium The Accelerating Colorectal Cancer Screening and Follow-up through Implementation Science (ACCSIS) Program. The overall aim of ACCSIS is to conduct multi-site, coordinated, transdisciplinary research to evaluate and improve CRC screening processes using implementation science strategies.

The second case study is Cancer Control Population Simulation for Healthcare Decisions (Cancer Control PopSim), a series of Centers for Disease Control and Prevention (CDC)-funded studies [22–26] using microsimulation [27] to estimate the projected population health impact and cost-effectiveness of evidence-based CRC screening interventions and health policy changes. In this case, the implementation strategy being employed is modeling and simulating change to motivate adoption of a range of EBIs [28]. This work is intended to support future implementation efforts by quality improvement staff, federal agency partners, providers, clinic administrative staff, and population health leadership. Our simulation modeling has been used to estimate EBI implementation costs and impact on the percent of the target population up-to-date with CRC screening, CRC cases and deaths averted, life-years gained, and long-term cost savings. For each case study, we share how the described methods are used to support implementation and sustainment planning across phases. Since these insights are context-dependent, we start by defining the context in which the work happens.

### Identifying the target population and context

Our case studies, like other implemented EBIs, are situated within specific populations and contexts. Economic evaluations require clear understanding of the EBI’s target population, and the context in which they will be reached. This includes the target population’s size, geographic location, level of risk, and sociodemographics, and the characteristics of the context that determine EBI reach and adoption. Researchers should be mindful of existing inequities, how implementation resources may vary to adequately address these disparities, and the extent to which implementation outcomes may improve or worsen these inequities. In low-resource settings and when serving marginalized populations, resource allocation for EBI implementation requires more thoughtful assessment [5]. Implementing the SCORE intervention, for example, focuses on CRC screening among CHC patients, who screen at relatively low rates [29, 30] and face unique barriers [31, 32]. Implementation strategies, such as adding staff (e.g., patient navigator) to deliver centralized services, were used to address patients’ resource needs, including financial and transportation barriers to undergoing follow-up colonoscopy, and to limit the burden placed on CHC staff. Without the added resources planned upfront to develop and support these strategies, the expected gains in CRC screening associated with investment in a multicomponent EBI may not be realized.

### Mixed methods

We selected a set of methods to describe how qualitative and quantitative approaches can be integrated to estimate EBI implementation costs and impact. Although not an exhaustive list of methods to support economic analyses, these methods include the approaches used in our two case studies, and which we believe can be used to understand complex systems. In addition to being used to quantify the resources needed for EBI implementation (as we do in this paper), these methods can also be considered implementation strategies on their own; for example, process flow diagramming can be used both as an implementation strategy to assess organizational readiness and as a tool to support and assess resources needed for other implementation strategies [28, 33]. We build on Powell and colleagues’ work [28] to provide a novel way of thinking about implementation strategies as systems science methods that can optimize implementation success. We consider our use of the included methods to be a mixed-method approach because we were intentional about using each method to inform and build on other methods. These methods can be bidirectional and interactive in diverse ways based on the underlying research question. Table 2 identifies the methods and describes how they can be used to specify the boundaries of EBI implementation (i.e., within the scope of implementation) and estimate implementation costs and benefits by EPIS phase. Below, we categorize the methods into three primary groups: (1) methods for eliciting stakeholder, patient, and caregiver input; (2) systems mapping and time-and-motion analysis; and (3) simulation and sensitivity/uncertainty analysis. We assume a decision has already been made to implement a complex EBI.

**Table 2:** Methods of economic-focused data collection and analysis by phase of implementation

	Exploration	Preparation	Implementation	Sustainment
**Economic Evaluation Methods**	*Selection of interventions and consideration of what implementation requires using prior literature and examples*	*Selection and tailoring of implementation strategies and the creation of tools to support planned intervention implementation and to guide subsequent data collection*	*Data collection and ongoing evaluation of intervention implementation*	*Evaluation of implementation and effectiveness outcomes and planning for local sustainment and/or scale-up/spread of the intervention*
Stakeholder interviews, surveys, and periodic reflections	• Identify key stakeholders and assess their readiness for an intervention under consideration • Review existing studies on personnel time and costs associated with intervention implementation • Solicit feedback on potential barriers to implementation of the intervention	• Assess capacity and stakeholders’ goals and preferences • Determine which activities and types of costs are associated with usual care • Design guides to evaluate costs and time from the perspective of those implementing the intervention • Identify and prepare for potential challenges in intervention implementation	• Obtain feedback on the successes and challenges of implementation • Understand unexpected challenges (e.g., turnover) in intervention implementation • Identify opportunities to better support agents involved in implementing the intervention • Assess how usual care and associated costs may change over time as a result of intervention implementation	• Assess stakeholder buy-in for sustainment of the intervention and necessary adaptations • Understand unexpected challenges (e.g., turnover) in intervention implementation
Patient and caregiver interviews, surveys, and focus groups	• Review existing studies on patient/caregiver preferences, costs, etc. when designing the intervention and identifying relevant costs	• Design guides to evaluate costs and time from the patient/caregiver perspective	• Conduct interviews/surveys to collect patient/caregiver costs and time spent • Refine implementation strategies to better address the needs of patients/caregivers and reduce burden	• Include patient/caregiver-level costs in societal perspective for cost-effectiveness analyses
Process flow diagramming	• Review process flow diagrams identified in the literature and compare documented processes to local context • Identify potential resource points • Consider which personnel are needed and what types of training they need • Identify potential adaptations to implementation given local challenges, processes in place, and available resources	• Assess key processes involved that affect costs before doing microcosting, cost-effectiveness analysis, etc. through developing a local process flow diagram • Identify all resource points • Consider which personnel are needed and what types of training they need • Obtain feedback on the diagram from diverse implementation agents to ensure the included steps are comprehensive and reflective of their activities • Map cost data collection activities, including type, source, and frequency, onto the steps to guide methods for estimating costs • Identify points to assess fidelity using the steps outlined in the diagram	• Track costs and other resources associated with each step involved in implementing the intervention • Identify and measure potential voltage drops (i.e., retention, missed opportunities, etc.) in implementation reach and desired outcomes • Revise diagram as needed to reflect changes and adaptations to the intervention and how it is implemented	• Include costs from all steps documented in the diagram in cost-effectiveness analyses • Adapt processes to address voltage drops and increase effectiveness in the local context • Identify resource-intensive process steps and consider opportunities for change • Track and evaluate costs associated with sustainment of the intervention
Time-and-motion analysis	• Obtain preliminary list of cost components from existing studies and consider feasibility in the local context	• Harmonize cost measures • Design time-and-motion study, including the creation of toolkits for tracking costs, using process flow diagrams • Pilot time-and-motion toolkits and solicit feedback from agents involved in implementation	• Observe processes and record activities • Assess at multiple time points to consider improved efficiencies over time, differences across staff, etc.	• Use time-and-motion data in cost-effectiveness and simulation studies • Determine how different inputs affect overall cost-effectiveness • Optimize how processes are performed • Estimate budget for those interested in program implementation in other settings
System support mapping	• Review existing studies on resources and staffing required for intervention implementation • Identify the individuals responsible for implementing the intervention in the local context	• Identify the individuals responsible for implementing the intervention in the local context • Develop guide for facilitating system support mapping sessions	• Identify roles and responsibilities of staff implementing the intervention and the resource requirements associated with those responsibilities • Consider contextual factors that may affect resource adequacy and delineation of roles • Adapt intervention as needed to address issues related to resource adequacy in the local context	• Adapt intervention as needed to address issues related to resource adequacy in the local context • Determine the essential activities and resources needed to implement the intervention to support scale-up and spread
Simulation	• Review prior simulation studies to motivate the implementation of an intervention • Develop and continue to update a list of the intervention costs and effectiveness estimates from literature review • Identify potential gaps in the types of costs associated with implementation of the intervention reported in prior studies	• Identify short-term and long-term outcomes of interest • Document all decisions made about how the intervention is implemented • Estimate the costs and other resources required to implement the intervention using tools developed (e.g., process flow diagrams, time-and-motion toolkits, etc.)	• Estimate the costs and other resources required to implement the intervention using tools developed (e.g., process flow diagrams, time-and-motion toolkits, etc.) • Develop and refine model that accounts for all aspects of intervention implementation in the local context • Estimate population-level projections of the impact of the intervention for the local context • Compare expected outcomes in the local context for the overall population and by subgroups to explore potential equity issues	• Develop and refine model that accounts for all aspects of intervention implementation in the local context • Estimate population-level projections of the impact of the intervention for the local context • Compare expected outcomes in the local context for the overall population and by subgroups to explore potential equity issues • Estimate the potential cost-effectiveness of the intervention if sustained or scaled up • Determine optimal strategies for intervention sustainment/scale-up of the intervention
Sensitivity/Uncertainty analysis	• Identify important parameters in prior estimates of intervention effectiveness and costs • Identify areas of uncertainty related to the intervention and implementation of the intervention in prior simulation studies	• Identify important parameters in prior estimates of intervention effectiveness and costs • Identify areas of uncertainty related to the intervention and implementation of the intervention in prior simulation studies • Identify areas of uncertainty while planning implementation of the intervention in the local context	• Identify areas of uncertainty while planning implementation of the intervention in the local context • Track variations in the time spent and costs associated with intervention implementation to inform best and worst case scenario estimates • Determine the key parameters and assumptions that have the largest influence on the results • Evaluate the impact of uncertainty on outcomes of interest in the local context • Estimate the sensitivity of the simulation results to changes in intervention implementation in the local context • Graphically display the results of sensitivity and uncertainty analyses to inform decision-making	• Determine the key parameters and assumptions that have the largest influence on the results • Evaluate the impact of uncertainty on outcomes of interest in the local context • Estimate the sensitivity of the simulation results to changes in intervention implementation in the local context • Estimate and report plausible ranges of outcomes of interest if the intervention is sustained or scaled up • Graphically display the results of sensitivity and uncertainty analyses to inform decision-making

#### Eliciting stakeholder, patient, and caregiver input

##### Stakeholder engagement methods

Economic evaluations should identify the appropriate analytic perspective (i.e., the point of view taken during analysis) and use it to determine which costs and benefits are measured [10, 34, 35]. Input from stakeholders (e.g., potential implementation agents, partner organizations, funders, etc.) is needed to consider the relevant costs and benefits associated with different courses of action, and whether and how EBI implementation and selected strategies will fit within their priorities and constraints. Stakeholder biases and preferences may affect the perceived usefulness of EBIs or implementation strategies [36]. For example, in a study about shared decision-making within cancer care, stakeholder interviews revealed widespread concerns about the likelihood of losing revenue as a substantial implementation barrier [6]. Thus, to successfully implement an EBI, especially a complex and resource-intensive intervention, stakeholder perspectives about the utility, feasibility, costs, and benefits of the intervention and implementation strategies must be incorporated across implementation phases.

The Exploration phase provides an opportunity to review stakeholder perceptions of intervention components and implementation strategies documented in prior literature and to engage context-specific stakeholders in discussions about EBI development. During the Preparation phase, interviews and surveys with diverse stakeholders can elicit their expectations and capacity for EBI implementation. These methods may also provide insight into what usual care or other implementation strategies entail in their local contexts to provide a comparator(s) for how the EBI is implemented. In the Implementation phase, these methods along with periodic reflections [37] can be used to solicit input on EBI implementation successes and challenges and to clarify resource use and unexpected or unintended expenses. Periodic reflections, in which agents are asked about their experiences with EBI implementation at multiple time points, can identify potential challenges and adaptation opportunities [37]. As examples, routine discussions with implementation agents may reveal time-consuming or otherwise resource-heavy steps threatening EBI sustainment, or provide information about a policy or contextual change directly impacting EBI implementation. Capturing this information allows for more accurately measuring resources expended and developing solutions, which can help to obtain stakeholder buy-in for EBI sustainment.

In the case of SCORE, during the Exploration phase, we met with state-level stakeholders (e.g., Colorectal Cancer Roundtable) to solicit input on the feasibility of candidate EBIs and fit with current workflows using local consensus discussions [28]. During Preparation, we engaged CHC and endoscopy providers in workgroups to identify resource barriers and facilitators to implementation [28]. The workgroups created process flow diagrams for each of SCORE’s central components (described later). During Implementation, we employed survey and interview methods to estimate resource use and to evaluate implementation agents’ perceptions of intervention implementation and its impact on usual care. These methods included (1) questionnaires about CHC screening processes in the absence of SCORE (e.g., who performs each activity, how frequently, and time spent per patient), (2) brief, electronic surveys assessing intervention acceptability mid-implementation (e.g., are intervention objectives clear?), and (3) semi-structured interviews about how intervention implementation has affected clinic work processes (e.g., how, if at all, has your work changed because of SCORE?). Insights from periodic reflections [37] are being used to proactively determine how to address any possible threats to sustainment and improve outcomes.

##### Patient and caregiver interviews, surveys, and focus groups

Patients are an important group whose time and costs incurred should be included in economic evaluations [35, 38]; however, patient costs are often excluded or incompletely assessed [38]. The overall cost-effectiveness of an implemented EBI can vary substantially when accounting for patient-level costs. In an economic evaluation of screening colonoscopy versus no screening, the cost per life-year saved with colonoscopy increased by 68% when patients’ time spent prepping for, undergoing, and recovering from the colonoscopy was costed [39]. Like patient costs, caregiver costs should also be tracked if relevant to the analytic perspective (for example, if a societal perspective is assumed). In the case of colonoscopy, a caregiver typically accompanies the patient, requiring additional time and other potential costs (e.g., time off work, childcare, etc.). Patient and caregiver interview or survey guides can be developed in the Preparation phase to map and understand patient-level resources and time required. Interviews and surveys can be conducted during the Implementation phase to prospectively track these resources, assess patient and caregiver burden, and adapt implementation strategies as needed. The costs estimated using these methods can then be included in analyses during the Sustainment phase.

The expected gains of implementing the SCORE intervention depend on patients being receptive to mailed FIT outreach and, if their results are abnormal, completing a follow-up colonoscopy. The implementation strategy of adding centralized staff to navigate FIT-positive patients to their diagnostic colonoscopy will only be successful if patients are willing to respond to the navigator and utilize the services offered. Therefore, we designed an interview guide for FIT-positive patients about their SCORE experiences that inquired about navigation and colonoscopy completion steps. We included a quantitative checklist for patients to report how long each step took (e.g., time spent driving to the pharmacy) and any out-of-pocket costs (e.g., bowel prep kit cost). We then qualitatively assessed which activities are most burdensome to patients and caregivers and how navigation may have alleviated these burdens. This mixed-method approach to estimating patient and caregiver resources will provide detailed cost data specific to the lower-resource CHC population targeted by SCORE. Additionally, it may help to identify ways to adapt, sustain, and/or scale existing implementation strategies to best meet patients’ needs and minimize their burdens.

#### Systems mapping and time-and-motion

##### Process flow diagramming and time-and-motion

Process flow diagramming (i.e., process mapping) is a method for visualizing the required steps in a process and areas for potential variation in pathways depending on the outcomes of certain steps [40, 41]. Process maps can aid in setting EBI boundaries during the Exploration and Preparation phases and using those boundaries to inform data collection in subsequent phases. Process mapping during the Preparation phase helps to document all steps involved in EBI implementation, identify resources required for those steps, and create mechanisms for tracking expended resources. During the Implementation phase, process maps of the intervention itself help to collect precise and comprehensive estimates of what it costs to implement the EBI, thus informing cost-effectiveness analyses. In the Sustainment phase, process maps allow teams to identify areas for improved efficiencies and develop strategies to institutionalize an EBI, such as translating resource requirements into staffing plans, job descriptions, and orientation plans for onboarding new staff.

Time-and-motion (TAM) analysis involves estimating labor-related inputs associated with EBI implementation [42]. Key processes involved in EBI implementation are assessed (for example by using process maps), toolkits are designed to track those activities, and identified processes are observed and recorded using the toolkits. This method allows for estimating the time required per activity, which can be used to estimate per-person labor costs. Conducting these observations at multiple time points allows for estimating differences in time (and thus costs) associated with specific activities across implementation agents and evaluating efficiencies over time. TAM data are an integral component of microcosting (i.e., bottom-up cost analysis) and can inform how to assign common resources that do not fit neatly into a single activity or category [43, 44]. Prior studies have demonstrated how related time-driven costing methods allowed for more accurate cost estimation of health interventions, including variation in delivery and associated costs across sites and personnel [45, 46]. Analysis of TAM data captures the total investment of personnel time and resources in EBI implementation and provides insight into how to optimize processes to support sustainment and scale-up.

**Fig. 1:**
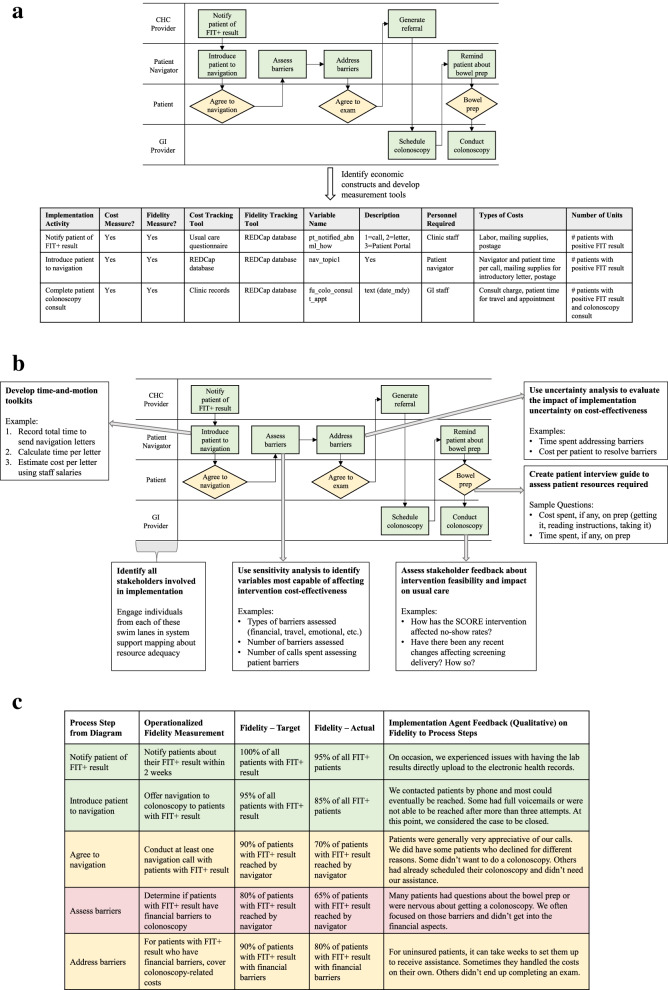
**a** Use of swimlane diagrams to identify economic and fidelity measures for the SCORE intervention during the Preparation phase. This is a simplified version of a process flow diagram for patient navigation to follow-up colonoscopy provided as part of the SCORE intervention. Examples are provided of how specific process steps are used to develop cost and fidelity measures and appropriate tools for measuring these constructs. *CHC* community health center, *FIT* fecal immunochemical test, *GI* gastrointestinal, *SCORE* Scaling Colorectal Cancer Screening Through Outreach, Referral, and Engagement. **b** Use of swimlane diagrams to inform mixed methods approach to estimating costs of implementing the SCORE intervention. This is a simplified version of a process flow diagram for patient navigation to follow-up colonoscopy provided as part of the SCORE intervention. For individual steps involved in implementing the patient navigation intervention, examples are provided for how diverse types of methods can be used to collect and estimate the required resources to implement that step. *CHC* community health center, *FIT* fecal immunochemical test, *GI* gastrointestinal, *SCORE* Scaling Colorectal Cancer Screening Through Outreach, Referral, and Engagement. **c** Example integration and presentation of mixed methods results. This is an example using hypothetical data of how we might integrate the quantitative results of our analysis (in this case, the proportion of patients who received each process step) with qualitative data from implementation agents. The color-coding is used to identify process steps from the process flow diagram included in (**a** and **b**) with low (< 70% of patients), moderate (between 70 and 84% of patients), and high (85% of patients or higher) fidelity. This structure can also be used to integrate cost estimates per step with qualitative findings

In the SCORE study, we used process mapping during the Preparation phase to develop the multicomponent intervention, plan for its implementation, and design our TAM analysis. Through consensus discussions with stakeholders [28], we developed “swimlane” process maps, which use lanes (i.e., rows) to delineate which agents perform specific steps and in which settings (e.g., CHCs, laboratories, mailed FIT outreach center). We used these diagrams to identify groups of activities requiring personnel time that could be observed in batches, such as mailing introductory letters. As with our other methods, we considered which steps are research-specific and which would need to be performed outside of the research context for programmatic success, including only the latter in our economic evaluation. For each activity, we developed a TAM costing tool to document the labor steps involved. For example, mailing introductory letters entailed identifying eligible patients, conducting mail-merges, labeling envelopes, and performing quality control. We piloted these tools during the Preparation phase and scheduled periodic observations during the Implementation phase. We used our swimlane diagrams during the Preparation phase to identify other non-labor costs associated with each step and to develop fidelity measures for tracking potential variations or adaptations in EBI implementation, which may have cost implications [12, 47]. For each process step, we documented how the associated costs and fidelity steps were to be measured, the frequency of data collection, and where to report the collected data (see Fig. 1a). In Fig. 1b, we demonstrate how we used these maps in combination with other methods to further develop our measurement tools. Figure 1c provides a hypothetical example of how these maps might be used to integrate quantitative and qualitative results to document gaps in fidelity along with implementation agents’ perception of burden incurred at each process step.

##### System support mapping

**Fig. 2:**
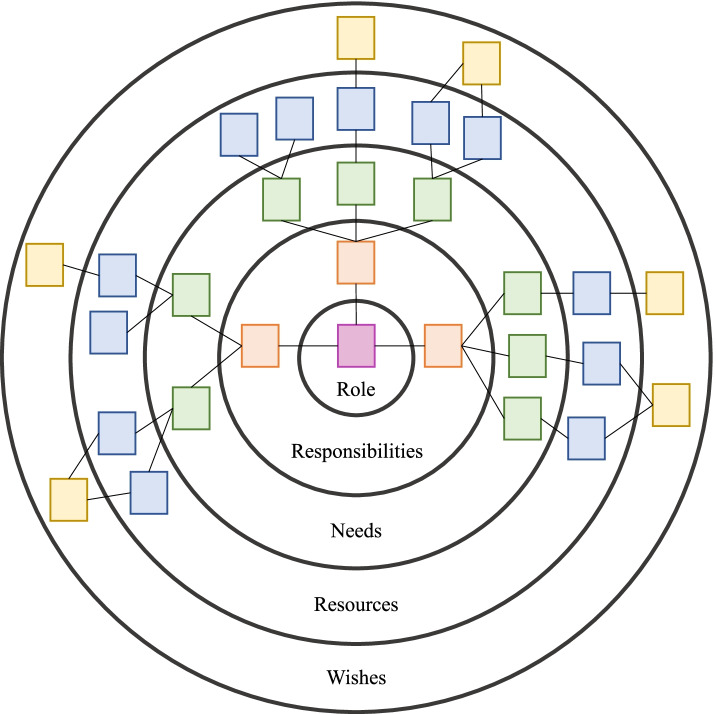
System support mapping (SSM) example. This is a stylized version of a system support mapping (SSM) diagram. In SSM sessions, each individual with a role in evidence-based intervention implementation reflects on each of the topics (e.g., role, responsibilities, etc.) listed in the rings. The squares represent individual notes or ideas per topic area and are connected across the rings to tell complete stories about each specific responsibility or task they undertake related to intervention implementation (each on its own orange square). To accomplish each responsibility or task, they are asked to name critical needs (green notes), resources they rely on to support those needs (blue notes), and, reflecting on how well those resources work, identify specific wishes for how they could be better supported in accomplishing that responsibility or task (yellow notes). Lines interconnect notes within a story about each named responsibility or task. The numbers of rings and notes per ring will vary across implementation agents and implementation studies. Maps can be made in person, with sticky notes, or virtually. In any case, each individual should verbally describe their map since this will enrich the documented map

System support mapping (SSM) is a structured systems thinking method that is used to elicit participants’ individual responsibilities in EBI implementation, primary needs and available resources to fulfill each responsibility, and quick reflection on resource adequacy [48]. Figure 2 provides an SSM example. SSM can be completed with individuals, or in a group—similar to a focus group where the facilitator guides participants through a structured assessment of their individual roles and resource adequacy to perform their roles. Previously used to learn how to support state and local maternal and child health professionals leading complex change initiatives [48], SSM aids in evaluating implementation of complex EBIs by understanding the experiences and perceptions of diverse agents. After identifying all individuals with an implementation role during the Preparation phase, SSM sessions can be conducted during the Implementation phase to collect structured agent feedback on specific activities undertaken by each to implement the EBI, critical needs to achieve each responsibility, specific resources used (with feedback on how useful they were), and suggestions for how to improve support for their implementation activities (perhaps including specific implementation strategies).

For the SCORE intervention, we invited CHC clinicians and administrative staff and quality improvement monitors involved in implementation process steps from the swimlane diagrams to participate in SSM sessions. The information gathered will help identify which agents are employing which implementation strategies and, thus, ensure all responsibilities and resources used are appropriately costed in the economic evaluation. Whereas our process maps detailed the specific steps being carried out by implementation agents, SSM allows for identifying possible redundancies, inefficiencies, or misunderstandings about EBI-related responsibilities individuals undertake, unexpectedly resource-heavy or under-supported activities, and further delineation of roles (e.g., which individual conducts each mailed FIT process step among the larger mailed FIT team). Agents’ recommendations for improvement can also be estimated in terms of their expected costs and benefits during the Implementation or Sustainment phases to inform decision-making about EBI adaptation and/or sustainment. Similarly, SSM may identify opportunities to streamline responsibilities or better support staff needs, which can be evaluated in the economic evaluation.

#### Modeling and sensitivity/uncertainty analysis

##### Simulation modeling

Simulation modeling can be used as an implementation strategy [28] to project the health and economic impacts of investment in EBIs. Simulation can help to adapt analyses to particular contexts to understand potential implementation gains and losses. For example, Medicaid enrollment is associated with high turnover and coverage gaps [49]. Using simulation, we can build in realistic assumptions about enrollee turnover, and thus more accurately analyze costs and benefits from the perspective of Medicaid decision-makers considering EBI implementation. In the Preparation and Implementation phases, steps for conducting simulation studies include identifying short-term and long-term outcomes of interest and estimating implementation costs using the aforementioned methods (TAM, stakeholder interviews, etc.). Findings from completed simulations can aid in making decisions about appropriate inputs and outputs. Models can also be used to monitor and provide feedback on implementation progress across sites to better reach implementation targets. During the Implementation and Sustainment phases, models can be developed to project the intervention’s population-level impact and cost-effectiveness in the local context and extrapolate these findings into the future or to other settings. Equity issues can be assessed by projecting outcomes for specific subgroups, such as those at increased risk of poor outcomes or for whom the implementation strategies may be inappropriate or infeasible. Distributional cost-effectiveness analysis [50, 51] and related methods for quantifying equity-efficiency tradeoffs [52] can help to understand and address health inequities. Results of equity-focused analyses can inform EBI sustainment in the local context, and the selection and adoption of EBIs and implementation strategies for other settings (i.e., motivating EBI implementation by other organizations during the Exploration phase).

Our Cancer Control PopSim work shows how simulation can be used to project the downstream impact of EBI implementation in higher-risk populations. Using our model, we estimated the cost-effectiveness of multiple EBIs for improving CRC screening and long-term CRC outcomes (e.g., cancers averted) in priority populations, such as African Americans [25], the uninsured [23, 24], and Medicaid enrollees [22–24]. Costs of EBI implementation, CRC screening and diagnostic procedures, and CRC treatment were included. Among Oregon Medicaid enrollees, for example, we found three of five EBIs simulated to be cost-effective compared to usual care if Medicaid decision-makers are willing to spend up to $230 per additional year up-to-date on CRC screening [22]. In North Carolina, we identified mailed reminders for Medicaid enrollees and mass media campaigns for African Americans as cost-effective EBIs, costing approximately $15 and $30, respectively, per additional life-year up-to-date on CRC screening [24]. We also showed that expansion of North Carolina’s Medicaid program would more substantially reduce CRC diagnoses among African Americans, compared to non-Hispanic Whites, and result in greater cost-savings over the long-term due to averted treatment costs [25]. These analyses allow for targeted EBI implementation planning by detailing the funds needed to efficiently address health inequities.

Related to SCORE specifically, our plans are to have a cost-effectiveness model focused on our target CHC population and with more detail on how the different intervention components and implementation strategies affect success at micro-level steps. The model can then help to project the downstream impacts associated with our outcomes (fidelity, reach, etc.) at each process step.

##### Sensitivity/uncertainty analysis

Estimation and simulation of EBI implementation costs and benefits are critical to evaluating whether investment in EBI implementation should continue. Yet, these decisions remain challenging due to uncertainty about the future and conflicting priorities. While the base-case economic evaluation (i.e., analysis using core model assumptions and most likely input values) may indicate that EBI implementation is cost-effective for improving outcomes, questions remain about under which circumstances this is true. The overall cost-effectiveness of EBI implementation may vary across agents, populations, settings, time horizons, analytic perspectives, model assumptions, and implementation outcomes (e.g., fidelity, reach). Sensitivity/uncertainty analysis can serve multiple purposes, such as estimating how important a particular variable is to the overall cost-effectiveness and understanding how results may change due to differing parameter values and structural assumptions across contexts [53]. These analyses can help stakeholders broaden their thinking about whether to implement or sustain an EBI from simply a yes/no decision using base-case estimates to considering the range of plausible estimates and assumptions that may affect decision-making.

Many types of sensitivity/uncertainty analyses can be conducted using the methods for estimating costs and benefits previously described. Examples include scenario analysis in which variation in conclusions is assessed using specific values for uncertain parameters; threshold analysis to identify the particular value(s) at which EBI implementation becomes or is no longer cost-effective; and probabilistic sensitivity analysis where multiple uncertain parameters are varied simultaneously using distributions of possible estimates [54]. Regardless of which analyses are conducted, areas of uncertainty related to further EBI implementation in the current setting or in other settings should be proactively identified (potentially through systems mapping and stakeholder engagement) as EBIs are planned and implemented. For example, capturing variations in TAM estimates by agent type and over time can provide ranges of estimates for conducting a best-case/worst-case analysis. Questions of most importance to decision-makers about sustaining cost-effective EBIs and implementation strategies should be prioritized; for example, depending on context and perspective, the outcomes of focus in an economic evaluation may vary and may not include patient-level utility estimates. Analytic prioritization could also include comparing different scenarios of how personnel, start-up funds, and other resources are allocated across EPIS phases and their relative impact on cost-effectiveness over time. Varying the analytic time horizon could also reveal important insights, such as how long EBIs may need to be implemented to achieve objectives. The impact of uncertainty on outcomes of interest and sensitivity of the results to changes in EBI implementation should be evaluated for the local context in the Implementation phase and to support long-term planning during the Sustainment phase.

Our Cancer Control PopSim model outputs demonstrate how sensitivity/uncertainty analysis can be used to consider the impact of tradeoffs related to EBI implementation. We used this model to evaluate the effectiveness of multicomponent CRC screening interventions in reaching national screening targets (Hicklin et al: "Assessing the impact of multicomponent interventions on colorectal cancer screening through simulation: what would it take to reach national screening targets?", in progress). We varied the percentage of the target population reached by each intervention, initially considering the differences in impact on selected outcomes assuming 25%, 50%, 75%, and 100% intervention reach levels. We then conducted a threshold analysis to determine which specific level of reach is needed to achieve screening targets under different circumstances. Our analysis demonstrated that the expected downstream effects of implementing EBIs are driven by multiple factors, including intervention effectiveness, intervention reach, implementation costs, and equity considerations (i.e., which subpopulations are targeted by which interventions). With our SCORE model, we will use data on implementation costs and outcomes associated with individual process steps, together with input from stakeholders, to explore how intervention operations and implementation strategies might be adapted to support sustainment. Using mixed methods to collect comprehensive and context-specific costs across implementation outcomes will aid greatly in having meaningful estimates to weigh tradeoffs over the short- and long-terms.

### Integration of methods

**Fig. 3:**
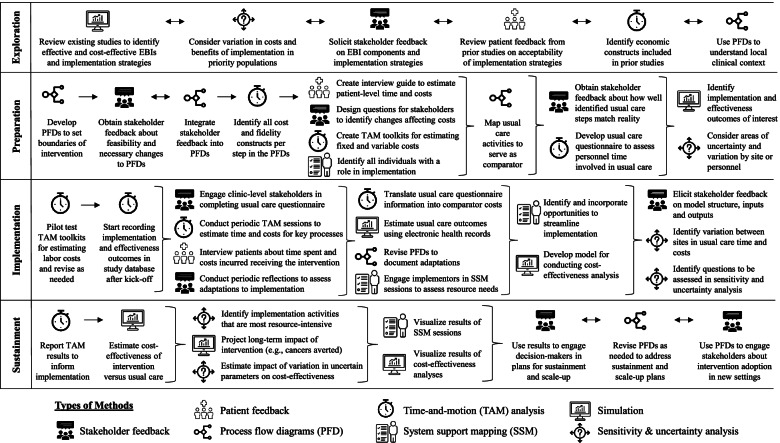
Example schematic for clarifying cost-related activities for economic evaluation of SCORE intervention across EPIS phases. This figure depicts how we integrated quantitative, qualitative, and systems approaches to estimate the costs and impact of implementing the SCORE intervention across implementation phases. Economic evaluations of other EBIs may vary considerably in the number and types of methods used, as well as how these methods are integrated, for multiple reasons (e.g., available resources, local context, intervention complexity, etc.). We included a highly detailed version to help inform planning for other economic evaluations. Bidirectional arrows indicate that the methods inform each other in a more cyclical process, and brackets indicate that multiple methods are being used simultaneously

The methods, described above, can be used individually to support economic evaluations of implementation research or, preferably, in combination with each other. Figure 3 provides a detailed schematic of our mixed-method approach to the SCORE economic evaluation. While it is not necessary for economic evaluations to include this level of complexity, our goal was to show how diverse methods can be used to inform each other across implementation phases when implementing complex EBIs. Below, we provide guidance on how to account for possible adaptations, and how to visualize data collected through economic evaluations—both of which are important to supporting decision-making about EBI implementation.

### Adaptation

Economic evaluations of implementation studies should seek to measure and optimize adaptation of EBI implementation [55]. As complex interventions are implemented, the interventions and implementation strategies are also being changed, potentially due to staff changes, lessons learned from earlier phases, environmental or policy changes, new evidence-based guidance, and changes in available resources. These adaptations may affect or be identified to improve EBI implementation costs and/or benefits. Thus, the monitoring of adaptations should be a continuous process across EPIS phases. Capturing stakeholder and implementation agent feedback, mapping process steps, using periodic reflections, and other mixed-method approaches are all useful for identifying and costing these changes.

### Visualizing economic outputs

**Fig. 4:**
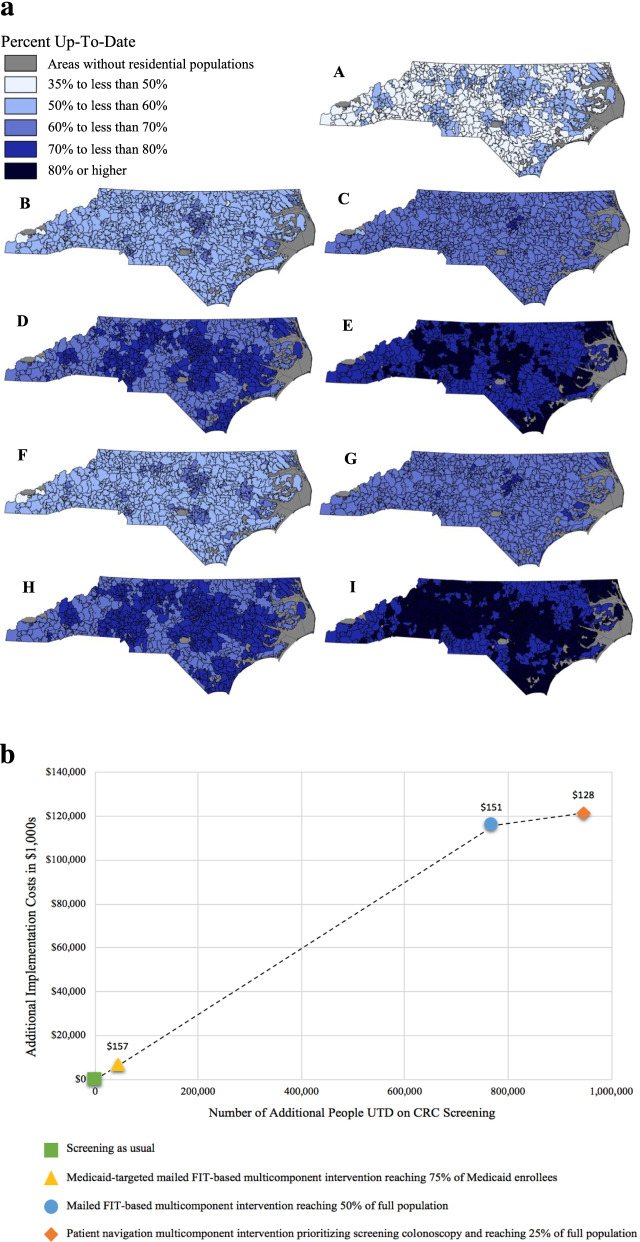
**a** Percent of eligible North Carolina residents up-to-date on CRC screening by zip code assuming different types of interventions, levels of intervention reach, and health insurance policy after 5 years of intervention. A: Status quo scenario (i.e., absence of intervention or health policy change). B: Implementation of mailed FIT-based multicomponent interventions, assuming 25% reach of eligible population and no Medicaid expansion. C: Implementation of multicomponent interventions prioritizing patient navigation to screening colonoscopy, assuming 25% reach of eligible population and no Medicaid expansion. D: Implementation of mailed FIT-based multicomponent interventions, assuming 75% reach of eligible population and no Medicaid expansion. E: Implementation of multicomponent interventions prioritizing patient navigation to screening colonoscopy, assuming 75% reach of eligible population and no Medicaid expansion. F: Implementation of mailed FIT-based multicomponent interventions, assuming 25% reach of eligible population and Medicaid expansion. G: Implementation of multicomponent interventions prioritizing patient navigation to screening colonoscopy, assuming 25% reach of eligible population and Medicaid expansion. H: Implementation of mailed FIT-based multicomponent interventions, assuming 75% reach of eligible population and Medicaid expansion. I: Implementation of multicomponent interventions prioritizing patient navigation to screening colonoscopy, assuming 75% reach of eligible population and Medicaid expansion. Maps can help to guide decision-making about where and how to best invest limited resources to improve health outcomes. These maps can help to assess the potential impact of various combinations of approaches for increasing CRC screening at the population level by region, all of which have important cost and resource implications. **b**. Value frontier based on multicomponent CRC screening intervention implementation costs over 5 years. This figure, which is shown for illustrative purposes, compares the incremental number of age-eligible North Carolina residents up-to-date (UTD) on CRC screening (*x*-axis) and the incremental implementation costs (*y*-axis) for multicomponent intervention scenarios after 5 years. The incremental cost-effectiveness ratios (ICERs) are reported for each scenario above the data point. Cost and effectiveness estimates are based on prior CRC screening intervention studies [20, 56–76]. Costs of screening tests and required follow-up are excluded. We assumed the level of reach that would be feasible for each intervention scenario. The target population for the scenarios includes all age-eligible state residents, except for one scenario which only reaches Medicaid enrollees

Visualizing data derived through economic evaluations is important for informing and reflecting on decision-making about EBI implementation. Figure 4 provides examples of how we displayed our Cancer Control PopSim data to guide mobilization of limited resources to achieve the greatest gains in CRC screening in North Carolina. We created maps displaying the expected change in CRC screening by zip code associated with 5-year implementation of multicomponent interventions (Fig. 4a). These maps indicate which regions are expected to most benefit from EBI implementation and which approaches (e.g., increasing reach versus changing intervention components) are likely to be most impactful. Then, we created a value frontier to report the cost per additional person up-to-date on CRC screening of our intervention scenarios (Fig. 4b), building on cost and effectiveness estimates from prior studies [20, 56–76]. Value frontiers help to report the cost per health outcome gained in cases where there is not an established willingness-to-pay threshold for determining cost-effectiveness. Decision-makers can identify which interventions are most cost-effective based on their established budget, or weigh the potential advantages or disadvantages of changing their budget. Visualization tools can be used and updated across EPIS phases as new data become available to guide which intervention(s) are adopted, how they are implemented, whether to adapt implementation strategies, and how best to extend implementation into new settings or over time.

## Conclusions

The use of multiple, diverse methods across implementation phases when conducting economic evaluations of complex interventions is important for setting boundaries, collecting rich, context-specific estimates of EBI implementation costs and downstream impacts, informing decisions about EBI investment, and understanding adaptations along the way. The visual display of data collected through these methods can further aid in weighing tradeoffs in how and where to invest limited resources.

We focused on estimating the cost and impact of complex EBIs and implementation strategies, and using this information to inform implementation and sustainment, across EPIS phases. The proposed methods can be supplemented with more traditional costing methods (e.g., microcosting), following best practice guidelines [77]. Economic evaluations of some simpler EBIs would also benefit from the described methods. For example, process maps depicting simpler EBIs may clarify the process steps and implementation agents. However, the effort involved in process flow diagramming may not be prudent if there are few swimlanes and/or process steps.

In our experience with SCORE and Cancer Control PopSim, combining the described methods provided more comprehensive data than we would have obtained using the methods in isolation. Additional research is needed to assess potential patterns in which methods work well together, in what order the methods should optimally be conducted, and which methods are most feasible given resource constraints. There may also be additional methods not described here that could contribute to the planning and execution of economic evaluations of implemented EBIs. Our scope was limited to understanding the value of integrating different types of methods within economic evaluations and providing an initial menu of methods and their functions to select from per implementation phase.

In some contexts, resources may not be available to implement comprehensive sets of economic methods. We encourage teams in these situations to, at a minimum, have staff dedicated to collecting and tracking implementation costs and benefits. Use of methods that can be integrated into existing work processes, such as periodic reflections, might be prioritized. We also emphasize that it is essential for funders to invest in resources needed to conduct economic evaluations in lower-resource settings—otherwise, there is a greater risk of continuing to implement and sustain sub-optional interventions or failing to learn about more cost-effective approaches.

The integration of quantitative and qualitative methods when estimating EBI implementation costs and benefits allows for more nuanced data collection and thoughtful considerations of how to efficiently and equitably support public health initiatives. By extending our analytic options for economic evaluations, we have an opportunity to improve the study of EBI implementation and subsequently, patient and societal outcomes.

## Data Availability

The datasets used and/or analyzed during the current study are available from the corresponding author on reasonable request.

## Abbreviations

ACCSIS: Accelerating CRC Screening and Follow-up through Implementation Science
PopSim: Population Simulation
CDC: Centers for Disease Control and Prevention
CHC: Community health center
CRC: Colorectal cancer
EBI: Evidence-based intervention
FIT: Fecal immunochemical testing
NCI: National Cancer Institute
SCORE: Scaling Colorectal Cancer Screening Through Outreach, Referral, and Engagement
SSM: System support mapping
TAM: Time-and-motion
